# Acupuncture and related therapies for insomnia symptoms in hypertensive patients: protocol for a network meta-analysis of randomized controlled trials

**DOI:** 10.3389/fneur.2026.1730432

**Published:** 2026-03-04

**Authors:** Ning Sun, Ying-peng Zhi, Ting Feng, Yun-jiao Sheng

**Affiliations:** 1 Rehabilitation Medicine Center and Institute of Rehabilitation Medicine, West China Hospital, Sichuan University, Chengdu, China; 2 Key Laboratory of Rehabilitation Medicine in Sichuan Province, West China Hospital, Sichuan University, Chengdu, China; 3 Department of Hypertension, Jinan Municipal Hospital of Traditional Chinese Medicine, Jinan, Shandong, China; 4 Department of Respiratory, Jinan Municipal Hospital of Traditional Chinese Medicine, Jinan, Shandong, China; 5 Department of Geriatrics, Jinan Municipal Hospital of Traditional Chinese Medicine, Jinan, Shandong, China

**Keywords:** acupuncture, complementary therapy, hypertension, insomnia, network meta-analysis

## Abstract

**Introduction:**

Insomnia and hypertension frequently co-occur and may exacerbate cardiovascular risk. While acupuncture and related therapies (ARTs) are widely used for sleep symptoms, their comparative effectiveness across modalities remains unclear. This protocol outlines a Bayesian network meta-analysis (NMA) to compare the effectiveness of ARTs for insomnia in adults with hypertension and inform clinical decision-making.

**Methods and analysis:**

This protocol follows preferred reporting items for systematic review and meta-analysis protocols (PRISMA-P) and is registered in PROSPERO. We will search PubMed, Embase, Cochrane Library (CENTRAL), Web of Science, and four Chinese databases (CNKI, Wanfang, VIP, and SinoMed) from inception to January 2026, and we will also screen WHO ICTRP, ClinicalTrials.gov, and ChiCTR without language restrictions. Eligible studies are randomized controlled trials enrolling adults with hypertension and insomnia, comparing an ART with sham, usual care, sleep hygiene, pharmacotherapy, or another ART. The primary outcome is change in global insomnia severity at post-treatment or at the first follow-up; secondary outcomes include sleep parameters, blood pressure, quality of life, mood, and adverse events. Pairwise meta-analyses will be performed using RevMan. A Bayesian random-effects NMA will be implemented in R (v4.4.1), with network plots and league tables generated in Stata (v15.1). The assumptions of transitivity and coherence will be assessed using design-by-treatment and node-splitting approaches; model fit will be evaluated using the deviance information criterion (DIC). Risk of bias will be assessed using RoB 2, and the certainty of evidence will be rated using GRADE adapted for NMA, with prespecified subgroup and sensitivity analyses.

**Registration number:**

PROSPERO; identifier CRD420251173289.

## Introduction

Hypertension and insomnia frequently co-occur (1) and may reinforce each other through shared biological pathways (2, 3). Patients with hypertension commonly report difficulty initiating or maintaining sleep, early-morning awakening, non-restorative sleep, and daytime dysfunction (3). Globally, an estimated 1.4 billion adults aged 30–79 years are living with hypertension (4). Sleep disturbance is highly prevalent in this population; 33.3% of 6,708 older hypertensive adults had poor sleep quality, and outpatient cohorts report prevalence between 68.4 and 78.8% (5–7). Prospective evidence indicates that insomnia accompanied by objectively short sleep is associated with higher odds of prevalent hypertension and nearly doubles the risk of incident hypertension (8). These associations are stronger in women (9). Poor sleep is also linked to worse blood-pressure control, reduced adherence to antihypertensive therapy, and elevated cardiovascular risk (10). Consequently, developing effective, safe, and scalable strategies to relieve insomnia symptoms specifically in hypertensive populations is an important clinical priority.

Current management of insomnia emphasizes non-pharmacological approaches, with cognitive behavioral therapy for insomnia (CBT-I) recommended as the first-line treatment (11, 12). Pharmacotherapies (e.g., benzodiazepine receptor agonists, sedating antidepressants, or melatonin-receptor agents) may also be considered. However, these medications can cause residual daytime sedation, cognitive impairment, falls, tolerance, and drug interactions (13, 14). Such concerns are particularly relevant for older adults with hypertension and polypharmacy (14). Access to CBT-I is often limited, and the majority of patients prefer complementary options with favorable safety profiles (15).

Acupuncture and related therapies (ARTs) are increasingly used to manage insomnia and cardiovascular symptoms (16, 17). ARTs can be grouped into invasive modalities, including manual acupuncture, electroacupuncture, and auricular acupuncture, and non-invasive modalities, including acupressure, auricular acupressure, transcutaneous electrical acupoint stimulation (TEAS), and moxibustion. Proposed mechanisms for sleep improvement include modulation of autonomic balance (18), alterations in melatonin and *γ*-aminobutyric acid signaling (19, 20), anti-inflammatory effects (19), and changes in cortical–subcortical networks involved in arousal and affect regulation (21). Previous studies have suggest that ARTs can reduce insomnia severity and improve sleep quality, but head-to-head comparisons among modalities are scarce (16, 17, 22). Recent studies have focused on hypertensive cohorts report clinically meaningful improvements on validated insomnia measures with acupuncture vs. usual care or sham, although small samples and methodological heterogeneity temper the certainty of these estimates (23).

Traditional pairwise meta-analysis cannot simultaneously compare multiple ARTs that have not been directly tested against each other (24). Network meta-analysis (NMA) integrates direct and indirect evidence to estimate comparative efficacy across a network of interventions and to generate treatment rankings. To address the evidence gap, we will conduct an NMA of randomized controlled trials (RCTs) evaluating ARTs for insomnia symptoms in adults with hypertension. Our objectives are to:

1. Synthesize randomized evidence to estimate the comparative effects of acupuncture and related therapies on insomnia symptoms in adults with hypertension.
2. Generate decision-quality evidence by ranking these interventions to inform clinical choice.

## Methods and analysis

### Study design and registration

Registration of this systematic review protocol has been completed in PROSPERO (CRD420251173289). We will report the protocol in line with the preferred reporting items for systematic review and meta-analysis protocols (PRISMA-P) to ensure transparent and complete reporting (25), with further information available in Supplementary File 1. Our methodological approach will follow established standards, drawing on the Cochrane Handbook for Systematic Reviews of Interventions for procedures and using the grading of recommendations, assessment, development and evaluation (GRADE) approach to assess the certainty of evidence. For the NMA, we will comply with PRISMA-NMA guidance (26), with the work planned to begin in January 2026 and conclude on 20 May 2026.

### Diagnostic criteria

Adults with a documented diagnosis of hypertension and concurrent insomnia will be eligible. Hypertension will be considered eligible if diagnosed in the original trial using clearly stated criteria consistent with major international guidelines, including the 2017 American College of Cardiology/American Heart Association guideline (ACC/AHA), the 2023 European Society of Hypertension guideline (ESH), or the National Institute for Health and Care Excellence guideline “Hypertension in adults: diagnosis and management” (NG136; NICE), based on office blood pressure, 24-h ambulatory blood pressure monitoring, or home blood pressure monitoring with explicit thresholds (27–29). Insomnia will be considered eligible if defined using established diagnostic standards, including the Diagnostic and Statistical Manual of Mental Disorders, Fifth Edition, Text Revision (DSM-5-TR), the International Classification of Sleep Disorders, Third Edition, Text Revision (ICSD-3-TR), or the International Classification of Diseases, 11th Revision (ICD-11), or equivalent trial-defined criteria requiring difficulty initiating or maintaining sleep or early-morning awakening, accompanied by daytime impairment and explicit frequency/duration thresholds (30–32).

### Eligibility criteria

1. *Types of participants*. Adults (≥18 years) with a documented diagnosis of hypertension and concurrent insomnia symptoms, as defined in the diagnostic criteria section.
2. *Types of interventions*. Acupuncture and related therapies are delivered as stand-alone or adjunctive treatments. We define ARTs as interventions that stimulate recognized acupoints or auricular points using needles, heat, pressure, or electrical stimulation delivered through their acupoints. For the primary network, each ART modality will be modeled as a distinct intervention node when sufficient evidence is available, including manual acupuncture, electroacupuncture, auricular acupuncture, body acupressure, auricular acupressure, TEAS, moxibustion, and acupoint catgut embedding.

To preserve clinical interpretability and transitivity, we will not merge modalities *a priori*. If the network is sparse or disconnected, we will apply a prespecified, rule-based merging strategy only to clinically similar modalities that share invasiveness and stimulation characteristics, and we will report all merging decisions transparently and test them in sensitivity analyses. Interventions must target recognized acupoints and report session frequency and duration. Trials will be excluded if they are purely pharmacological or herbal, involve acupoint injection with drugs, use non-acupoint neuromodulation, involve massage or Tuina without an acupuncture component, or employ multi-component packages where the acupuncture effect cannot be isolated.

For multi-component interventions (e.g., acupuncture combined with moxibustion), we will handle them as follows: (i) if the combination constitutes a distinct and recurring regimen across trials, we will treat it as a separate combination node (e.g., “manual acupuncture + moxibustion”); (ii) if combinations are infrequent or heterogeneous, we will include them only when the co-intervention structure is balanced across the trial arms, and we will explore their influence via sensitivity analyses. Trials in which the specific contribution of ARTs cannot reasonably be isolated will remain excluded.

1. *Types of comparators*. Eligible comparators include sham or placebo controls, wait-list controls, usual care, sleep hygiene education, pharmacotherapy, cognitive behavioral therapy for insomnia, or another acupuncture-related modality. Co-interventions are permitted if they are balanced across arms.
2. *Types of outcomes*. The primary outcome is change in global insomnia severity from baseline to post-treatment or first follow-up, assessed with validated instruments and extracted using a prespecified hierarchy. Eligible measures include the Insomnia Severity Index (ISI), Pittsburgh Sleep Quality Index (PSQI), Athens Insomnia Scale (AIS), and other validated global insomnia/sleep-quality scales. When multiple instruments are reported, outcomes will be extracted according to a predefined priority order: ISI, followed by PSQI, then AIS, and finally other validated scales. Secondary outcomes include diary or objective sleep parameters, blood pressure outcomes (primary: office blood pressure; secondary: 24-h ambulatory blood pressure metrics, including daytime/nighttime blood pressure and nocturnal dipping when reported), quality of life, mood symptoms, and adverse events.
3. *Types of studies*. Study types included include RCTs, including individually randomized, cluster-randomized, and crossover designs with first-period data. No restrictions on intervention dose or duration. No language restrictions.

### Exclusion criteria

We will exclude all non-randomized designs, case series or case reports, qualitative or modeling studies, editorials, opinions, letters, protocols without results, conference abstracts without full data, and secondary research such as narrative or systematic reviews, meta-analyses, or umbrella reviews. Studies will be excluded when sleep complaints are primarily due to conditions requiring specific therapy. Trials lacking an acupuncture-related therapy arm or not allowing the effect of acupuncture to be isolated, including purely pharmacological or herbal interventions, acupoint injection with drugs, non-acupoint neuromodulation, or massage/tuina without an acupuncture component, will be excluded. Records with insufficient outcome data for the prespecified primary endpoint, after unsuccessful attempts to obtain missing information from authors or duplicate/overlapping publications, will be excluded, with the most complete dataset retained. Non-human or preclinical studies are not eligible.

### Search strategy

We will systematically search eight bibliographic databases, from inception through January 2026, including PubMed, the Cochrane Library (CENTRAL), Embase, Web of Science Core Collection, China National Knowledge Infrastructure (CNKI), Wanfang Data, VIP (Chinese Scientific Journals Database), and SinoMed (CBM). To mitigate publication bias, we will also screen trial registries (WHO ICTRP, ClinicalTrials.gov, and the Chinese Clinical Trial Registry), and we will manually search reference lists of included studies and relevant reviews. No language restrictions will be applied; equivalent Chinese keywords will be used in Chinese databases.

Search strategies will combine controlled vocabulary (e.g., MeSH/Emtree) and free-text terms across three concepts: ① Target conditions: hypertension/high blood pressure and insomnia/sleep disturbance; ② Acupuncture and related therapies: e.g., acupuncture, electroacupuncture, auricular acupuncture, acupressure/auricular acupressure, transcutaneous electrical acupoint stimulation, moxibustion, and acupoint catgut embedding; and ③ Randomized trial design: randomized/randomised, controlled clinical trial. Full, database-specific strategies are provided in Supplementary File 2.

### Study selection and data extraction process

**Figure 1:**
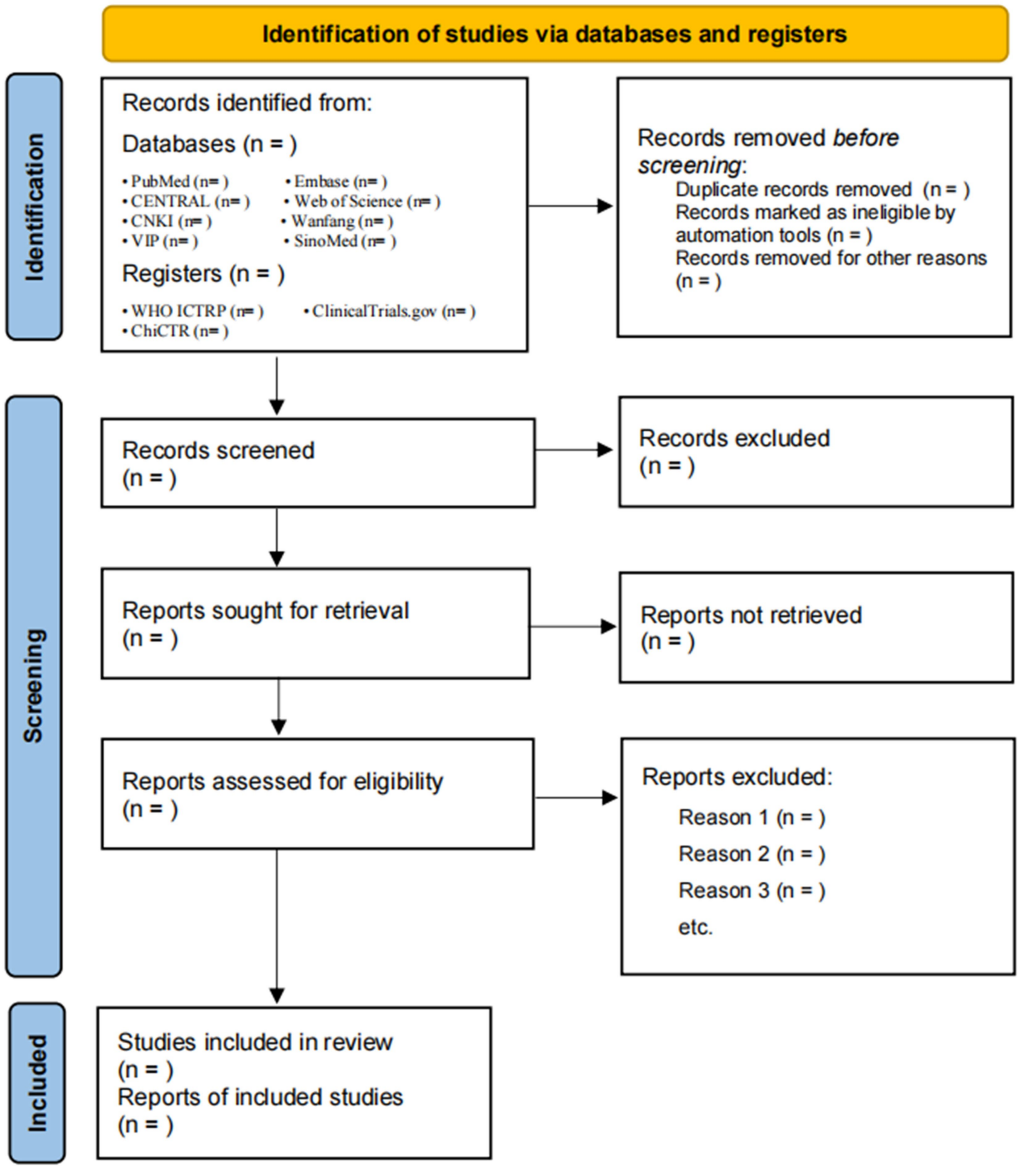
Flow diagram of literature screening.

Records will be de-duplicated in EndNote X9. Two reviewers (Y-pZ and TF) will independently screen titles/abstracts and then full texts, against eligibility criteria; disagreements will be resolved by consensus or a third reviewer. Reasons for full-text exclusions will be recorded, and the process is summarized in a PRISMA flow diagram (Figure 1).

Data will be extracted independently and in duplicate using a standardized form, which will include:

1. Study identifiers and design.
2. Participant characteristics (age, sex, insomnia severity/duration, hypertension stage, and key comorbidities).
3. Intervention/comparator details (acupuncture modality, acupoints/parameters, dose, delivery, and co-interventions). In addition to intervention modality, we will extract intervention-specific parameters to characterize treatment “dose” and intensity, including acupoint selection and prescription principles, session duration, frequency, total number of sessions, treatment duration, and, where applicable, stimulation parameters (e.g., electrical frequency/intensity for electroacupuncture/TEAS and moxibustion type and exposure time). These variables will be used to assess clinical heterogeneity and to support prespecified subgroup analyses and, when feasible, network meta-regression.
4. Outcomes/time points—primary: change in global insomnia severity from baseline to end of treatment or first follow-up; secondary: sleep parameters, blood pressure, quality of life, mood, and adverse events/withdrawals.
5. Numerical data to compute effect sizes (means, SD/SE, change scores, event counts, and numbers analyzed) and attrition. To ensure consistent directionality, all insomnia scales will be coded so that higher scores indicate worse insomnia; where necessary, scores will be reverse-coded. Change-from-baseline data will be extracted preferentially. In addition, we will extract prespecified potential effect modifiers relevant to transitivity, including baseline blood-pressure control/status (controlled vs. uncontrolled), insomnia characteristics (severity and duration), baseline and concurrent use of hypnotic medications, screening/reporting of comorbid sleep disorders (particularly obstructive sleep apnea), intervention dose and intensity (sessions per week, total sessions, and treatment duration), sex distribution, age, key comorbidities, comparator type, and co-intervention structure.

For missing or unclear information, corresponding authors will be contacted; studies will be excluded if essential data remain unavailable after two attempts over 14 days.

### Quality assessment

We will assess the risk of bias with the Cochrane RoB 2 tool at the outcome level (33). The primary endpoint is the change in global insomnia severity at the first post-treatment assessment. Two reviewers (Y-pZ and TF) will work independently after calibration. Five domains are evaluated: randomization process, deviations from intended interventions, missing outcome data, measurement of the outcome, and selection of the reported result. Each domain and the overall judgment will be rated as low risk, with some concerns, or high risk.

For the randomization process, a low risk of bias required an adequate sequence and robust allocation concealment (e.g., computer-generated list plus centralized allocation or sequentially numbered, opaque, sealed envelopes). Quasi-random methods or unconcealed allocation indicated a high risk. For deviations from intended interventions, lack of blinding raised concern only when it plausibly altered adherence or co-interventions, and when analyses were not conducted according to the intention-to-treat principle. For missing data, substantial or unbalanced attrition without appropriate handling increased risk. For outcome measurement, unblinded assessors using subjective scales increased concern, whereas validated procedures and blinded assessment reduced it. For selective reporting, we will compare reported outcomes and time points with protocols or registries.

Disagreements are resolved by consensus or third-reviewer adjudication. We will document justifications and sources. Graphical summaries will be produced in RevMan 5.4. Sensitivity analyses will exclude studies at high risk of bias.

### Pairwise meta-analysis

We will perform conventional pairwise meta-analyses in RevMan 5.4 for all direct head-to-head comparisons. Effect measures will be standardized mean differences for insomnia scales, mean differences for blood pressure outcomes, and risk ratios for dichotomous endpoints. Heterogeneity will be quantified with *Q*, *I*^2^, and *τ*^2^; model choice (fixed vs. random effects) will follow Cochrane guidance (34). We will explore outliers and influences using leave-one-out analyses. When ≥10 studies inform a contrast, small-study effects will be assessed using funnel plots and appropriate tests. Multi-arm trials will be handled by splitting shared comparators or by using methods that account for the correlation of effect estimates to avoid double-counting. Pairwise estimates will serve as the benchmark for the subsequent network meta-analysis and will help appraise the assumptions of similarity and transitivity; if the evidence network is sparse or disconnected, the pairwise results will constitute the primary synthesis (26).

### Network meta-analysis

We will perform a Bayesian random-effects NMA using R (v4.4.1) with the gemtc package and summarize network geometry and league tables in Stata (v15.1), following PRISMA-NMA guidance (26). Continuous outcomes will be synthesized as mean differences (MD) when the same scale is used or as standardized mean differences (SMD) when instruments differ; dichotomous outcomes will use risk ratios (RR), all reported with 95% credible intervals (CrI). Models will be contrast-based with a common between-study heterogeneity standard deviation (*τ*) across comparisons. For relative treatment effects, we will use weakly informative normal priors *d_k_* ~ N(0, 10^2^) for all non-reference treatments. For heterogeneity, we will use a half-normal prior: *τ* ~ HN(0, 1). Multi-arm trials will be modeled within likelihood to account for correlation and avoid double-counting. We will depict network geometry and report a contribution matrix. We will assess the network connectivity and geometry prior to synthesis. If the evidence network is sparse or disconnected, we will restrict the primary NMA to the largest connected component and report disconnected components separately. For interventions or comparisons that cannot be linked within a connected network, we will present conventional pairwise meta-analyses as the primary synthesis. Where clinically justified, we will consider prespecified, rule-based node-merging strategies to improve connectivity and will examine the impact of such decisions in sensitivity analyses.

Assumptions will be examined via transitivity checks on prespecified effect modifiers and coherence via global design-by-treatment tests and local node-splitting; where relevant, loop-specific inconsistency factors will be presented. We will assess the plausibility of transitivity by comparing the distribution of these prespecified effect modifiers across treatment comparisons and summarizing them in tables. Where sufficient data are available, we will explore heterogeneity related to these modifiers through prespecified subgroup analyses and, when feasible, network meta-regression. Model fit will be compared using the deviance information criterion (DIC) and posterior residual deviance. Relative rankings will be summarized using the surface under the cumulative ranking curve (SUCRA) and displayed as rankograms, interpreted alongside effect sizes, Credible intervals (Crls), heterogeneity, and coherence diagnostics. In sensitivity analyses, we will assess robustness to alternative heterogeneity priors, including *τ* ~ HN(0, 0.5) and *τ* ~ Uniform(0, 2).

### Subgroup analysis and sensitivity analysis

We will explore heterogeneity using prespecified subgroups. When ≥10 trials contribute to a covariate, we will fit random-effects network meta-regression with study-level moderators; otherwise, we will perform stratified NMAs. Planned subgroups include baseline insomnia severity (subthreshold vs. clinical), hypertension control at baseline (controlled vs. uncontrolled), sex (proportion female ≥60% vs. <60%), screening for primary sleep disorders (obstructive sleep apnea screening reported vs. not reported), comparator type (sham/attention vs. usual care vs. pharmacotherapy vs. cognitive behavioral therapy for insomnia), treatment class (invasive vs. non-invasive), treatment dose (≥3 sessions per week or ≥12 total sessions vs. lower dose) and duration (≥4 weeks vs. <4 weeks), geographical setting, and overall risk of bias (low or some concerns vs. high). Where data permit, we will further explore dose- and intensity-related heterogeneity using reported stimulation parameters (e.g., electrical stimulation frequency/intensity for electroacupuncture/TEAS) in network meta-regression or stratified analyses. We will report interaction estimates, residual heterogeneity, and clinical plausibility, while acknowledging the ecological nature and interpretative limits of study-level subgroup analyses (35, 36).

We will test robustness by excluding studies at high risk of bias; excluding trials without explicit screening for obstructive sleep apnea; using endpoint scores instead of change scores; excluding studies with imputed variances or converted summary statistics; varying modeling assumptions (fixed-effect vs. random-effects; alternative weakly informative priors for the heterogeneity SD); removing influential studies via leave-one-out analyses; and excluding small studies (total sample <50 or <30 per arm). We will compare effect estimates, heterogeneity (*τ*), coherence diagnostics, and rankings across scenarios and interpret differences for decision relevance.

### Assessment of publication bias

Small-study effects will be assessed using comparison-adjusted funnel plots (Stata 15.1, netfunnel) Analyses will be performed only for outcomes with ≥10 trials.

### Evidence quality assessment

We will rate certainty with GRADE (37) adapted for network meta-analysis. Risk of bias will use RoB 2 at the outcome level. We will downgrade if high-risk studies contribute materially. Heterogeneity will use *I*^2^ in pairwise analyses. In the network, we will use between-study SD and predictive intervals. We will downgrade for substantial, unexplained variability. Indirectness will be judged when populations, interventions, comparators, outcomes, or indirect evidence diverge from the review question. Imprecision will be judged by the 95% interval between minimally important differences and the required information size. Incoherence will be assessed through local checks and, when appropriate, global tests. We will downgrade if the inconsistency persists. Publication bias will be explored with comparison-adjusted funnel plots and registry cross-checks. We will downgrade when small-study effects or selective reporting are suspected. All judgments will be documented with concise justifications.
