# Supplementary material for: Acupuncture and related therapies for insomnia symptoms in hypertensive patients: protocol for a network meta-analysis of randomized controlled trials
Source: Front Neurol. 2026 Mar 4;17:1730432. doi: 10.3389/fneur.2026.1730432 (PMC12996139; doi:10.3389/fneur.2026.1730432)
Supplement: Supplementary file 2 [file Table_2.DOCX]

**Search strategies of this study.**

**1.PUBMED**

1#Acupuncture[MeSH Terms] OR Acupuncture Points[MeSH Terms] OR Acupuncture, Ear[MeSH Terms] OR Acupuncture Analgesia[MeSH Terms] OR Acupuncture Therapy[MeSHTerms] OR Auriculotherapy[MeSH Terms]

2#Acupuncture*[Title/Abstract] OR Needling[Title/Abstract] OR

Electroacupuncture*[Title/Abstract] OR Electro-acupuncture[Title/Abstract] ORAcupoint Therapy[Title/Abstract] OR Acupuncture Treatment[Title/Abstract] OR Acupuncture Treatments[Title/Abstract] OR Needle Therapy[Title/Abstract] OR silver needle[Title/Abstract] OR moxibustion[Title/Abstract] OR de qi[Title/Abstract] OR meridian[Title/Abstract] ORAuriculotherapy[Title/Abstract] OR needle pricking[Title/Abstract] OR needling[Title/Abstract] OR fire needle[Title/Abstract] OR fire needling[Title/Abstract] OR three-edged needle[Title/Abstract] OR blood letting Therapy[Title/Abstract] OR pricking blood therapy[Title/Abstract] OR Needle Warming Therapy[Title/Abstract] OR scalp acupuncture[Title/Abstract] OR auricular acupuncture[Title/Abstract] OR ear acupuncture[Title/Abstract] OR intradermal needling[Title/Abstract] OR acupoint embeddingtherapy[Title/Abstract] OR acupoint application[Title/Abstract]

3# 1# OR 2#

4#Hypertension[MeSH Terms] OR Hypertension[Title/Abstract] OR High Blood Pressure*[Title/Abstract]

5# 3# AND 4#

6#Sleep Initiation and Maintenance Disorders[MeSH Terms] OR Disorders of Initiating and Maintaining Sleep[Title/Abstract] OR DIMS [Title/Abstract] OR Sleeplessness[Title/Abstract] OR Insomnia[Title/Abstract] OR Early Awakening [Title/Abstract] OR Sleep Initiation Dysfunction[Title/Abstract]

7# 5# AND 6#

8# Randomized Controlled Trial[Publication Type] OR Randomized Controlled Trials as Topic[MeSH Terms] OR Randomized Controlled Trial[All Fields] OR RCT[All Fields] OR Trial*[All Fields] OR random*[Title/Abstract]

9# 7# AND 8#

**2.EMBASE**

#1 'Acupuncture'/exp

#2 'Acupuncture Points'/exp

#3 'Acupuncture, Ear'/exp

#4 'Acupuncture Analgesia'/exp

#5 'Acupuncture Therapy'/exp

#6 'Auriculotherapy'/exp

#7 'acupuncture*':ti,ab,kw OR 'electroacupuncture*':ti,ab,kw OR 'electro-acupuncture':ti,ab,kwOR 'acupoint therapy':ti,ab,kw OR 'Acupuncture Treatment':ti,ab,kw OR 'Acupuncture Treatments':ti,ab,kw OR 'needle therapy':ti,ab,kw OR 'silver needle':ti,ab,kwOR'moxibustion':ti,ab,kw OR 'de qi':ti,ab,kw OR 'meridian':ti,ab,kw OR 'auriculotherapy':ti,ab,kwOR 'needle pricking':ti,ab,kw OR 'needling':ti,ab,kw OR 'fire needle':ti,ab,kw OR'fire needling':ti,ab,kw OR 'three-edged needle':ti,ab,kw OR 'blood letting therapy':ti,ab,kwOR'pricking blood therapy':ti,ab,kw OR 'needle warming therapy':ti,ab,kw OR 'scalp acupuncture':ti,ab,kw OR 'auricular acupuncture':ti,ab,kw OR 'ear acupuncture':ti,ab,kwOR'intradermal needling':ti,ab,kw OR 'acupoint embedding therapy':ti,ab,kw OR 'acupoint application':ti,ab,kw

#8 #1 OR #2 OR #3 OR #4 OR #5 OR #6 OR #7

#9 ' Hypertension '/exp

#10 ' Hypertension ':ti,ab,kw OR ' High Blood Pressure*':ti,ab,kw

#11 #9 OR #10

#12 ' Sleep Initiation and Maintenance Disorders '/exp

#13 ' Disorders of Initiating and Maintaining Sleep ':ti,ab,kw OR ' DIMS ':ti,ab,kw OR ' Sleeplessness ':ti,ab,kw OR ' Insomnia ':ti,ab,kw OR ' Early Awakening ':ti,ab,kw OR ' Sleep Initiation Dysfunction ':ti,ab,kw

#14 #12 OR #13

#15 #11 AND #14

#16 'randomized controlled trial'/exp OR 'randomized controlled trial (topic)'/exp ORrandom*:ti,ab,kw OR 'RCT':ti,ab,kw OR Trial*:ti,ab,kw

#17 #15 AND #16

**3.COCHRANE DATABASE**

#1 MeSH descriptor: [Acupuncture] explode all trees

#2 MeSH descriptor: [Acupuncture Points] explode all trees

#3 MeSH descriptor: [Acupuncture, Ear] explode all trees

#4 MeSH descriptor: [Acupuncture Analgesia] explode all trees

#5 MeSH descriptor: [Acupuncture Therapy] explode all trees

#6 MeSH descriptor: [Auriculotherapy] explode all trees

#7 (acupuncture*):ti,ab,kw OR (electroacupuncture*):ti,ab,kw OR (electro-acupuncture):ti,ab,kwOR (acupuncture*):ti,ab,kw OR (electroacupuncture*):ti,ab,kw OR (electro- acupuncture):ti,ab,kw OR (acupoint therapy):ti,ab,kw OR (Acupuncture Treatment):ti,ab,kwOR(Acupuncture Treatments):ti,ab,kw OR (needle therapy):ti,ab,kw OR (silver needle):ti,ab,kwOR(moxibustion):ti,ab,kw OR (de qi):ti,ab,kw OR (meridian):ti,ab,kw OR (auriculotherapy):ti,ab,kw OR (needle pricking):ti,ab,kw OR (needling):ti,ab,kwOR(fire needle):ti,ab,kw OR (fire needling):ti,ab,kw OR (needle warming therapy):ti,ab,kwOR(scalpacupuncture):ti,ab,kw OR (auricular acupuncture):ti,ab,kw OR (ear acupuncture):ti,ab,kwOR(intradermal needling):ti,ab,kw OR (acupoint embedding therapy):ti,ab,kw OR (acupoint application):ti,ab,kw

#8 #1 OR #2 OR #3 OR #4 OR #5 OR #6 OR #7

#9 MeSH descriptor: [Hypertension] explode all trees

#10 (Hypertension):ti,ab,kw OR (High Blood Pressure*):ti,ab,kw

#11 #9 OR #10

#12 MeSH descriptor: [Sleep Initiation and Maintenance Disorders] explode all trees

#13 Disorders of Initiating and Maintaining Sleep:ti,ab,kw OR DIMS :ti,ab,kw OR Sleeplessness:ti,ab,kw OR Insomnia:ti,ab,kw OR Early Awakening :ti,ab,kw OR Sleep Initiation Dysfunction:ti,ab,kw

#14 #12 OR #13

#15 #11 AND #14

#16 MeSH descriptor: [Randomized Controlled Trial] explode all trees

#17 MeSH descriptor: [Randomized Controlled Trials as Topic] explode all trees

#18 (random*):ti,ab,kw OR (RCT):ti,ab,kw OR (Trial*):ti,ab,kw

#19 #16 OR #17 OR #18

#20 #15 AND #19

1. **WEB OF SCIENCE**

#1 TS=(Acupuncture)) OR TS=(Acupuncture Points) OR TS=(Acupuncture, Ear) ORTS=(Acupuncture Analgesia) OR TS=(Acupuncture Therapy)) OR TS=(Auriculotherapy)

#2TS=(acupuncture*) OR TS=(electroacupuncture*) OR TS=(electro-acupuncture) ORTS=(acupoint therapy) OR TS=(Acupuncture Treatment) OR TS=(Acupuncture Treatments) ORTS=(needle therapy) OR TS=(silver needle) OR TS=(moxibustion) OR TS=(de qi) ORTS=(meridian) OR TS=(auriculotherapy) OR TS=(needle pricking) OR TS=(needling) ORTS=(fire needle) OR TS=(fire needling) OR TS=(three-edged needle) OR TS=(blood lettingtherapy) OR TS=(pricking blood therapy) OR TS=(needle warming therapy) ORTS=(scalpacupuncture) OR TS=(auricular acupuncture) OR TS=(ear acupuncture) OR TS=(intradermal

needling) OR TS=(acupoint embedding therapy) OR TS=(acupoint application)

#3 #1 OR #2

#4 TS=(Hypertension) OR TS=(High Blood Pressure*)

#5 TS=(Disorders of Initiating and Maintaining Sleep) OR TS=(DIMS) OR TS=(Sleeplessness) OR TS=(Insomnia) OR TS=(Early Awakening) OR TS=(Sleep Initiation Dysfunction)

#6 #3 AND #4 AND #5

#7 TS=(Randomized Controlled Trial) OR TS=(Randomized Controlled Trials as Topic) ORTS=(Randomized Controlled Trials as Topic) OR TS=(random*) OR TS=(RCT) ORTS=(Trial*)

#8 #6 AND #7

1. **CNKI**

(针灸 + 针刺 + 电针 + 穴位 + 经络 + 得气 + 头皮针 + 激光针灸 + 耳针 + 耳穴 + 经皮穴位电刺激 + 指压 + 艾灸 + 埋线 + 火针 + 三棱针 + 放血 + 温针 ) * (高血压 + 原发性高血压 + 继发性高血压 + 高收缩压 + 高舒张压 + 血压) * (失眠 + 睡眠障碍 + 入睡困难 + 不寐 + 夜间觉醒 + 早醒)

1. **万方**

主题：（（“针灸”or “针刺 ”or “电针 ”or “ 穴位 ”or “ 经络 ”or “ 得气 ”or “ 头皮针 ”or “ 激光针灸 ”or “ 耳针 ”or “ 耳穴 ”or “ 经皮穴位电刺激 ”or “ 指压 ”or “ 艾灸 ”or “ 埋线 ”or “ 火针 ”or “ 三棱针 ”or “ 放血 ”or “ 温针 ”）and（“高血压”or “原发性高血压”or “继发性高血压”or “高收缩压”or “高舒张压”or “血压”）and（“失眠”or “睡眠障碍”or “入睡困难”or “不寐”or “夜间觉醒”or “早醒”））

1. **维普**

1. 针灸 + 针刺 + 电针 + 穴位 + 经络 + 得气 + 头皮针 + 激光针灸 + 耳针 + 耳穴 + 经皮穴位电刺激 + 指压 + 艾灸 + 埋线 + 火针 + 三棱针 + 放血 + 温针

2. 高血压+原发性高血压+继发性高血压+高收缩压+高舒张压+血压

3. 失眠+睡眠障碍+入睡困难+不寐+夜间觉醒+早醒

4.#1+#2+#3

1. **SinoMed**

（1）"针灸"[常用字段:智能] OR "针刺"[常用字段:智能] OR "电针"[常用字段:智能] OR "穴位"[常用字段:智能] OR "经络"[常用字段:智能] OR "得气 "[常用字段:智能] OR "头皮针"[常用字段:智能] OR "激光针灸 "[常用字段:智能] OR "耳针 "[常用字段:智能] OR " 耳穴"[常用字段:智能] OR "经皮穴位电刺激 "[常用字段:智能] OR "指压 "[常用字段:智能] OR " 艾灸 "[常用字段:智能] OR " 埋线 "[常用字段:智能] OR " 火针 "[常用字段:智能] OR " 三棱针 "[常用字段:智能] OR " 放血 "[常用字段:智能] OR " 温针

（2）"高血压"[常用字段:智能] OR "原发性高血压"[常用字段:智能] OR "继发性高血压"[常用字段:智能] OR "高收缩压"[常用字段:智能] OR "高舒张压"[常用字段:智能] OR "血压"[常用字段:智能]

（3）"失眠"[常用字段:智能] OR "睡眠障碍"[常用字段:智能] OR "入睡困难"[常用字段:智能] OR "不寐"[常用字段:智能] OR "夜间觉醒"[常用字段:智能] OR "早醒"[常用字段:智能]

（4）("失眠"[常用字段:智能] OR "睡眠障碍"[常用字段:智能] OR "入睡困难"[常用字段:智能] OR "不寐"[常用字段:智能] OR "夜间觉醒"[常用字段:智能] OR "早醒"[常用字段:智能]) AND ("高血压"[常用字段:智能] OR "原发性高血压"[常用字段:智能] OR "继发性高血压"[常用字段:智能] OR "高收缩压"[常用字段:智能] OR "高舒张压"[常用字段:智能] OR "血压"[常用字段:智能]) AND ("针灸"[常用字段:智能] OR "针刺"[常用字段:智能] OR "火针"[常用字段:智能] OR "电针"[常用字段:智能])
